# The roots of polarization in the individual reward system

**DOI:** 10.1098/rspb.2023.2011

**Published:** 2024-02-28

**Authors:** Germain Lefebvre, Ophélia Deroy, Bahador Bahrami

**Affiliations:** ^1^ Crowd Cognition Group, Ludwig Maximilian Unversität, Gabelsbergerstr 62, Munich 80333, Bavaria, Germany; ^2^ Philosophy, LMU, Geschwister Scholl Platz 1, Munich 80539, Bavaria, Germany

**Keywords:** polarization, confirmation bias, reinforcement learning, motivated reasoning, affective polarization

## Abstract

Polarization raises concerns for democracy and society, which have expanded in the internet era where (mis)information has become ubiquitous, its transmission faster than ever, and the freedom and means of opinion expressions are expanding. The origin of polarization however remains unclear, with multiple social and emotional factors and individual reasoning biases likely to explain its current forms. In the present work, we adopt a principled approach and show that polarization tendencies can take root in biased reward processing of new information in favour of choice confirmatory evidence. Through agent-based simulations, we show that confirmation bias in individual learning is an independent mechanism and could be sufficient for creating polarization at group level independently of any additional assumptions about the opinions themselves, *a priori* beliefs about them, information transmission mechanisms or the structure of social relationship between individuals. This generative process can interact with polarization mechanisms described elsewhere, but constitutes an entrenched biological tendency that helps explain the extraordinary resilience of polarization against mitigating efforts such as dramatic informational change in the environment.

## 1. Introduction

In an era marked by unprecedented connectivity and intense debates on critical issues ranging from climate change to political ideologies, unravelling the dynamics and determinants of opinion polarization is essential for grasping the complexities of modern societal discourses. Opinion polarization is usually distinguished from affective polarization, whereby members of different parties increasingly dislike each other [1–4], yet can still become a threat to democracy by preventing dialogue and consensus [5,6]. Its spread is observed on all continents [7] and has expanded partly due to the explosion of digital social media [8,9] where unlimited production and easy transmission of (mis)information are ubiquitous [10,11]. Diverse generative mechanisms as well as mathematical and computational models have been proposed to explain polarization [12–18], notably in terms of both micro*-* and macro-level characteristics of human social groups such as the architecture of social networks, the dynamics of interactions between individuals or the way those individuals process information (see [19,20] for recent and synthetic overviews of these mechanisms). Here, we offer a minimal, micro*-*level theory that starts from a robustly observed phenomenon in human individual behaviour and extrapolates to demonstrate polarization at the aggregate, social level without requiring any assumptions at macro-level such as network architecture, interaction dynamics or content. As explained further below, this detachment does not mean that the mechanism is the only basis of polarization, but that it is orthogonal to other mechanisms.

In *Micromotives and Macrobehaviour* [21] first published in 1978, Thomas Schelling introduced the lay reader to what formal economic models had revealed about the relation between the behaviour of individuals and the nontrivial emergent characteristics of the crowd that those individuals comprised. In our work, we follow a similar formalism. Recent studies showed that individual human decisions are biased towards confirmatory information even at very low cognitive levels, such as in visual perception [22,23] or in probabilistic reward learning [24–26]. In reinforcement learning tasks, we tend to take more into account any information that confirms the choice we just made and discard any information that goes against it, even when this choice is not intrinsically better than any other we could have made. Crucially, making the choice ourselves frames the subsequent feedback processing in favour of confirmatory information [26]. The bias dissipates if we observe and match another agent's decisions. These confirmatory tendencies have been hypothesized to relate to the noisy nature of decision making in humans. Given this peculiar property, confirmation bias has been shown to be reward maximizing in various learning environments through an extremization of options' value [27]. When considered in the context of reinforcement learning, confirmation bias is thus anchored in our reward system, rather than our belief formation and updating system [28], and it biases information processing simply by reinforcing previous choices. In broader terms, confirmation bias here is seen as shaping attitudes or value-based decisions rather than opinions.

Confirmation bias usually covers a set of related reasoning biases (see [29] for overview) which are recognized as contributing to polarization, notably in the context of motivated reasoning [30,31]. Here we examine confirmatory processing at a more basic cognitive level. Indeed in our study, polarization emerges from agents reinforcing their attitudes through unconscious decision patterns associated to biased value updating. Agents in our study do not require any consciously accessible belief or model of their environment for polarization to emerge at group level. Confirmation bias has been described as a mechanism of polarization decades ago [32] and various computational studies have subsequently implemented this process in different models [33–35]. Our model stands out by not requiring any interactions between agents for polarization to emerge, as well as describing mechanisms rooted in the human reward system. It combines reinforcement learning with a confirmation bias mechanism, and does not require any assumptions about social interactions nor network architecture for polarization to emerge.

Reinforcement learning has been recently introduced to the study of opinion dynamics with an innovative model explaining polarization through learning from social feedback [19]. This model stands out by introducing a simple learning mechanism to explain polarization without assuming bounded confidence [36] or negative influence [37]. The present study builds on these findings and extends the original model with a reward system accounting for confirmation bias—consistent with behavioural and neuroimaging studies [24–26,38]. Our model stands out by allowing polarization to emerge without any requirements about the group structure. Similarly to [19], it is the individual act of attitude expression itself that is reinforced and that leads here to the biased update of agents attitudes. This expression of attitude is then crucial in two main ways: it underlies the formation of attitudes and activates confirmation bias towards one or the other pole. To extrapolate the aggregate impact of individual learning confirmation bias on collective behaviour, we extend the reinforcement learning environment to the population level. We simulate a population of isolated individual agents who repeatedly make a statement about their attitude about two different conceptual objects. After every statement, they receive some (randomly sampled) valenced information with which they adjust their attitude according to a reinforcement learning update rule that incorporates confirmation bias. The conceptual objects can be thought of as any target of evaluation—from abstract political ideas, specific policies, brand affinities, or even scientific theories or medical recommendations. In our formulation here, they are neutral by definition and made equal in value to one another. We first show that polarization at the group level is a direct consequence of aggregating the behaviour of agents receiving new pieces of information and processing them in a confirmatory manner. Second, we emphasize that agent heterogeneity has varying effects on this phenomenon, depending on it being implemented in terms of initial preferences or in terms of confirmation bias intensity. We then demonstrate how polarizationis amplified by various macro*-*level phenomena observed in daily online life such as echo chambers and filter bubbles. Finally, we show that the polarization that arises from confirmation bias is resilient to dramatic changes in the environment: even the introduction of irrefutable evidence that entirely discredits one side of the controversy cannot eradicate such polarization in the short run.

## 2. Results

### (a) The virtual environment

**Figure 1.**
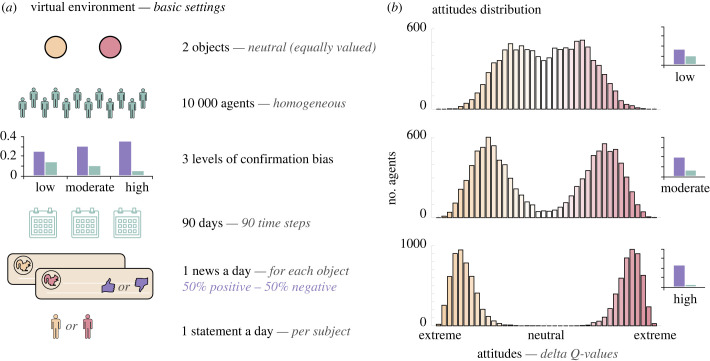
Attitude polarization in the simple environment. (*a*) Virtual environment parameters. The images introduce simulations parameters of the virtual environment in the basic version. (*b*) Attitude distributions. The histograms represent the distributions of the attitudes (i.e. the difference in individual Q-values) of agents at the end of the simulation, for three different confirmation bias levels. Colours represent the extremeness of the preferences.

In the first results sub-section, we detail the virtual environment used to perform our simulations (see also figure 1*a*). We simulated the behaviour of 10 000 indistinguishable agents. Each of them, at each time step, expresses their preference towards one of the two conceptual objects (e.g. political views) and receives a positive or negative piece of information for each of them (i.e. argument in favour or not of the objects) represented numerically by either −1 or 1, and randomly sampled for each agent.

Agents then update their attitudes towards the conceptual objects accordingly. The update process follows a variant of a Q-learning model allowing unequal treatment of confirmatory and disconfirmatory information and explaining accurately subjects’ behaviour in reinforcement learning tasks (a description of the model can be found in [25]). Attitudes are represented by the model Q-values, the attitude towards the favoured object (i.e. selected at time *t*) is updated such that:

$$Q_{\mathrm{chosen}}^{t+1}=Q_{\mathrm{chosen}}^{t}+\{\begin{array}{c} \alpha_{c}*PE,\;if\;PE>0\\ \alpha_{d}*PE,\;if\;PE<0 \end{array},$$

while the attitude towards the unfavoured object (i.e. not selected at time *t*) is updated such that:

$$Q_{\mathrm{unchosen}}^{t+1}=Q_{\mathrm{unchosen}}^{t}+\{\begin{array}{c} \alpha_{d}*PE,\;if\;PE>0\\ \alpha_{c}*PE,\;if\;PE<0 \end{array},$$

where $\alpha_{c}$ is the learning rate for confirmatory information and $\alpha_{d}$ is the learning rate for disconfirmatory information. Consequently, positive information concerning the chosen object and negative information concerning the unchosen object are considered confirmatory. On the other hand, negative information relative to the chosen object and positive information relative to the unchosen object are considered disconfirmatory. The prediction errors (PE) for each object (chosen or unchosen) is computed at each time step as:

$$PE_{\mathrm{object}}^{t}={\mathrm{Information}}_{\mathrm{object}}^{t}-Q_{\mathrm{object}}^{t},$$

where *Information* can be in favour or not of the object and equal to 1 or −1. Thus $PE_{\mathrm{chosen}}^{t}$ is the PE for the chosen object and used for factual learning. $PE_{\mathrm{unchosen}}^{t}$ is the PE for the unchosen object and is used for counterfactual learning. We defined three levels of confirmation bias: low bias with *α_c_* = 0*.*25 and *α_d_* = 0*.*15, moderate bias with *α_c_* = 0*.*3 and *α_d_* = 0*.*1 and high bias with *α_c_* = 0*.*35 and *α_d_* = 0*.*05. Unbiased learning is implemented such that *α_c_* = *α_d_* = 0*.*2. Learning rates in the moderate bias condition are based on the average learning rates fitted in humans in [26] while low and high biases learning rates are defined from the moderates ones with respectively a decrease or an increase of the difference between the two. In the original experimental study [25], average learning rates fitted in humans are even more extreme and would lead to an even greater amount of polarization. In humans, level of confirmation bias has been recently linked to affective states and information processing styles [39] as well as dopaminergic genes [40]. The decision is made through a softmax policy, allowing the presence of noise in the decision process. Extensive comparisons of attitude polarization with different learning rates combinations and decision noise can be found in electronic supplementary material (electronic supplementary material, figure S1 and figure S2 respectively).

Time steps *t* are thought as days and agents receive one piece of information, positive or negative, for each object, once a day. The probability of the information to be positive (versus negative) is 0.5, such that, on average, no object is more often supported than the other. Each day, each agent also expresses their attitude by making a statement about which object they favour based on their preferences regarding the two objects. The basic setting simulates arbitrarily 90 days.

### (b) Attitude polarization from individual confirmation biases

Results of the simulations in the basic environment without interaction and purely resulting from an aggregation of individual behaviours are represented on figure 1*b*. Histograms show the attitude distributions at the end of the time period considered (in the basic environment *t* = 90, simulations with 10^3^ epochs are presented in the supplementary materials, electronic supplementary material figure S5) for the three different levels of bias. A bimodal distribution indicates polarization in the population. While the group is not clearly polarized when agents account for new information with a low confirmation bias (top panel), polarization increases with the strength of their confirmation bias (middle and bottom panels). The high confirmation bias environment leads to a reduced diversity in attitude strength and the formation of two distinct groups. Additional simulations (electronic supplementary material, figure S1) show that an incremental increase of the bias is associated with a smooth and progressive transition from moderation to polarization.

### (c) Heterogeneous initialization

In this subsection, we explore the impact of heterogeneous initialization, in terms of both initial attitudes and confirmation bias levels. This initialization can be linked to any life event or situation that could have happened to the agents before beginning their journey with their attitude here (e.g. education, social-economic status, life events, etc.). For instance, religiosity is a strong predictor of attitude towards the theory of evolution [41]. Concerning the initial preferences, agents attitudes' value is not initialized to 0 as in previous simulations, but randomly drawn from the *Q*-values’ scale [−1,1] and made symmetrical between the two objects (e.g [0.31, −0.31]). As for the confirmation bias heterogeneity, the virtual population is divided in four bins, each of them associated with one level of the confirmation bias described above (*None*, *Low*, *Moderate* and *High*).

**Figure 2.**
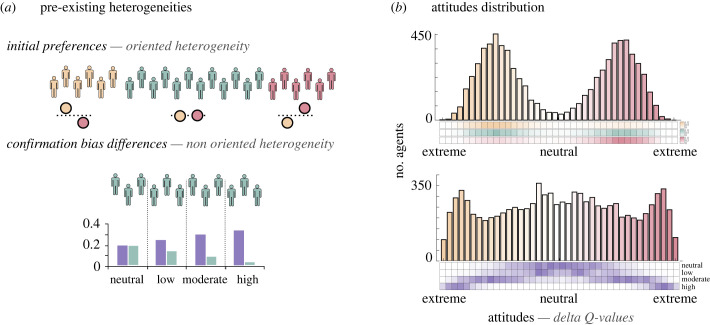
Attitude polarization in heterogeneous populations. (*a*) Agents' heterogeneity. Two types of heterogeneity are considered here. Heterogeneity in terms of initial attitudes (top panel) and heterogeneity in terms of confirmation bias strength. (*b*) Attitude distributions. Histograms represent the distributions of the attitudes (i.e. the variability in individual Q-values) of agents at the end of the simulation, with heterogeneous initial preferences (top panel) and heterogeneous confirmation bias levels (bottom panel). Colours vividness represents the extremeness of the attitude. Heatmaps represent the distributions of the attitudes of agents grouped according to their initial attitudes (top panel) or confirmation bias levels (bottom panel).

Heterogeneous initial attitudes and confirmation bias levels have different effect on the final attitude distribution. While initial attitudes play a role in the selection of the object that will be favored by agents and thus their side on the attitude spectrum (figure 2*b*, top panel), confirmation bias level governs the extremeness of agents' attitude but not their side on the attitude spectrum (figure 2*b*, bottom panel). Overall, agents with an initial preference for the yellow or the red object will be more likely to maintain and strengthen this attitude throughout the 90-day period (figure 2*b*, top panel, yellow and red heatmaps), while agents with neutral initial preferences will be equally distributed over the two (figure 2*b*, top panel, green heatmap). When heterogeneity is introduced through different confirmation bias strengths, it has no oriented effect towards one of the two objects but produces progressively more extreme attitudes with the increase in confirmation bias strength (figure 2*b*, bottom panel, purple heatmap).

### (d) Echo chambers and filter bubbles

**Figure 3.**
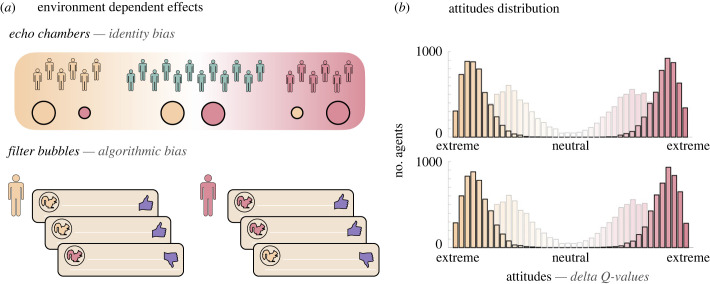
Attitude polarization in online networks. (*a*) Information transmission disruptions. In echo chambers mechanism, agents in a social network are more strongly and frequently connected to homogeneous others in terms of attitudes. Consequently, they received preferentially confirmatory information from others. In filter bubbles, algorithmic strategies exploit the agents' style of information consumption and feed them confirmatory information to retain them in the network. (*b*) Attitude distributions. The histograms represent the distributions of the attitudes (i.e. the difference in individual *Q*-values) of agents at the end of the simulation, when echo chambers effects (top panel) and filter bubbles effects (bottom panel) are implemented. Faded-colour histograms represent the distributions of the attitudes without any disruption in the information transmission (as seen figure 1*b*, middle panel). Colours represent the extremeness of the attitudes.

Results from the previous subsections are unequivocal. Confirmation bias alone has the ability to generate and maintain a substantial level of attitude polarization in a group of agents. Now we show that this phenomenon is further increased by implementing social interactions between agents, particularly of the type occurring when agents communicate online. We consider the impact of information exposition whether it comes from in-group dynamics (echo chambers) or algorithmic biases (filter bubbles) (figure 3*a*). Echo chambers have been defined as ‘environments in which the opinion, political leaning, or belief of users about a topic gets reinforced due to repeated interactions with peers or sources having similar tendencies and attitudes’ [32]. Filter bubbles refer to ‘the cumulative effects of personalization algorithms selectively choosing users' content, or what they will and will not see, based on their user profile and preferences’ [42]. Ultimately, in our environment as in real life, echo chambers and filter bubbles have a very similar effect (i.e. an increase of the probability of being exposed to confirmatory information and an associated decreased probability of being exposed to discomfirmatory information). This asymmetric exposure, be it due to an algorithmic selection of preferred information or a homogeneous social network, amplifies the polarization, both in terms of speed and intensity, that arises from confirmation bias in isolated agents.

Echo chambers (figure 3*a*, top panel) are implemented through an increase of the probability of getting a positive news about the preferred object and a symmetric decrease of this probability for the non-preferred object. This probability adjustments occur in agents having consistent attitudes for a fixed number of days (in the present case, 8) which make them *members* of the echo chamber. The probability increase is proportional to the number of members in the chamber at time *t*. Filter bubbles (figure 3*a*, bottom panel), in our environment, have a similar effect, that is an adjustment of the probability of receiving confirmatory news. However, the probability increase does not depend on other agents' attitudes but only on the individual proportion of statements in favour of the preferred object. From a fixed number of time periods (here 8), needed by the algorithm to learn from users’ preferences, the probability of being delivered a confirmatory information is adjusted to reflect this proportion. Agents' preferences then shape their information delivery in a more direct and individual way. In our simulations, echo chambers and filter bubbles have a very similar effect on the attitude distributions (figure 3*b*). In both cases, attitudes are more extreme and insulated from each other with the implementation of the social mechanism (versus without i.e. pale bars in figure 3*b*). The selective exposure to news confirming favoured objects then amplifies the impact of confirmation bias on the phenomenon described in previous sections. These news are still favourably taken into account and are also now more frequent.

### (e) Attitude polarization resists breakthrough information

**Figure 4.**
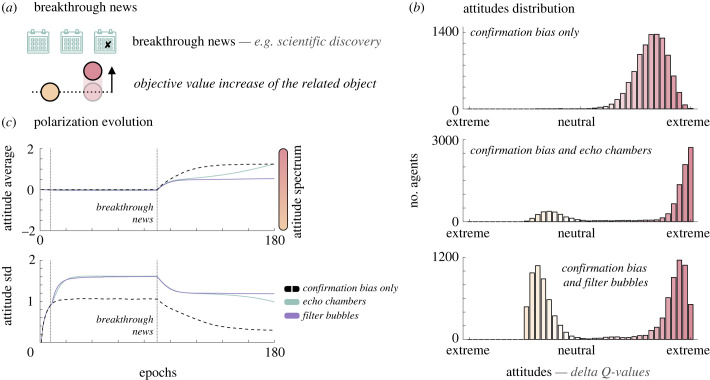
Attitude polarization resilience. (*a*) Breakthrough information. The images introduce the implementation of breakthrough news in the simulation. 180 days are now simulated and a breakthrough information is delivered to the world at time *t* = 90. At that time, the outcome values of the now objectively incontestable object become [0, 1] instead of [−1, 1], increasing its expected value. (*b*) Attitude distributions. The histograms represent the distributions of the attitudes (i.e. the difference in individual Q-values) of agents at the end of the simulation, when a breakthrough information is delivered at time *t* = 90 in three different scenarios: confirmation bias only, confirmation bias and echo chambers, confirmation bias and filter bubbles. (*c*) Polarization evolution. Line plots represent the evolution of the population's attitude towards one object (0 indicating no preference) and the evolution of attitudes standard error, used as a measure of polarization. Colours represent the three different scenarios simulated. Vertical dotted lines represent days threshold for echo chambers and filter bubbles (*x* = 8) and the onset of the breakthrough information (*x* = 90).

The strength of the confirmation bias effect described above is such that it can prevent extremist groups from changing their mind even if breakthrough information were to come out in the virtual environment (figure 4*a*). Breakthrough information or ‘external shocks’ [43] are defined as a sudden change in environmental information that will exogenously shift the attitude distribution towards one pole. Real life illustrations of this phenomenon could be the attitude towards vaccines during the COVID-19 pandemic, that may evolve after being exposed to increasing death toll news; or attitude towards climate change each time a natural disaster happens. In our environment, this breakthrough event occurs after a stable period (i.e. 90 time steps) where both objects are objectively equally valued. To implement the breakthrough, we increase of the expected value of one of the two contested object (i.e. the red object in figure 4*a*). Information received relative to this object cannot be negative anymore and outcome associated to it are now [0,1] (instead of the previous [−1, 1]) while the other object retains its previous value. This breakthrough has clearly favoured one object over the other and opting for the other one is now suboptimal.

If any population tested globally shifts their attitude towards the highervalued object, extremism remains on the other side of the attitude spectrum when echo chambers or filter bubbles are implemented (figure 4*b*) or when at least some agents in the population have a high level of confirmation bias (electronic supplementary material, figures S3 and S4). In the case of a population only affected by a moderate level of confirmation bias (figure 4*b*, top panel), there is no extremism left in the alternative side of the attitude spectrum. Attitudes are normally distributed and only the left tail of the distribution is close to neutrality. On the contrary, when echo chambers are implemented (figure 4*b*, middle panel), the polarization remains extremely high and the extremist group, even though reduced in size, still shares an attitude far from the otherwise widely adopted one. In the case of filter bubbles (figure 4*b*, bottom panel), the extremist group remains extremely important in size and polarization is also at a very high level despite a continuum of attitudes that maintains between the two extremities of the spectrum. Attitude polarization then is resilient to dramatic changes in the environmental information when a moderate confirmation bias joins forces with online phenomena such as echo chambers or filter bubbles. Furthermore, in the case where some or all agents in the population have instead a high level of confirmation bias, polarization can be persevered even without the consideration of these phenomena (see electronic supplementary material, figure S3B, top panel for a population constituted only of highly biased agents and electronic supplementary material, figure S4B, top panel for a heterogeneous population where a fourth of them are highly biased).

To analyse the impact of breakthrough information on the polarization dynamics, we computed two different variables at each time step, the mean and the standard deviation of the opinion distribution. The first is used as an indication of the overall attitude tendency in the population and is equal to 0 when no object is favoured overall, negative when the yellow object is favoured and positive when the red object is favoured. The second is used as a measure of polarization [44,45] with a higher value indicating more polarization. As expected, in all three cases, the average attitude shifted towards the red object (i.e. the difference in *Q*-values increased) after the breakthrough information onset (figure 4*c*, top panel). This shift was smaller when filter bubbles were implemented (figure 4*c*, top panel, purple line) due to the large group still supporting the alternative opinion. In the two other cases, *confirmation bias only* and *confirmation bias and echo chambers*, the shift was similar by the end of the 180 days but the trajectories were different. In the second case (figure 4*c*, top panel, green line), the echo chamber of the red object has progressively grown after the breakthrough information onset while the echo chamber of the yellow object has shrunk in the same time, amplifying continuously the shift towards the red object. The evolution of the standard deviation (figure 4*c*, bottom panel) confirms the previous analysis. Polarization progressively decreased after the breakthrough information delivery in all three cases but remained strong in the *echo chamber* and *filter bubble* cases (figure 4*c*, bottom panel, green and purple lines respectively). As for the mean, the continuous decrease of the standard deviation in the *echo chamber* case is due to the increase in size of the red object echo chamber and associated decrease in size of the yellow object echo chamber. An analysis of the distributions' evolution beyond 180 trials is presented in supplementary materials (electronic supplementary material, figure S6 and S7).

## 3. Discussion

By combining agent-based simulations with reinforcement learning, we showed that polarization can arise as a byproduct of statistically aggregating the individual behaviours of isolated agents who process information regardless of content in a confirmatory way. The stronger the confirmation bias, the more polarized the attitudes distribution. This polarization is amplified in speed and extent by social and algorithmic phenomena observed online such as echo chambers and filter bubbles. The latter's ultimate effect is an increase of the probability with which agents receive confirmatory feedback, catalysing the confirmation bias effect on the extremization of attitudes. Finally, we showed that once polarized, a socially connected population of such self-confirming agents resists the introduction of breakthrough discrediting information that makes one of the two objects rationally untenable.

Remarkably, a population of disconnected agents that follow a simple, biologically plausible learning algorithm would tend to polarize even though in their environment, options have the same intrinsic value. The extent of the polarization depends then solely on the intensity of the confirmation bias assumed. The results of the present study are therefore orthogonal to the questions of the existence of echo chambers and filter bubbles, and other higher level cognitive processes at play in information processing and the more sophisticated social interaction mechanisms. While the latter are undoubtedly of importance in the study of opinion polarization, low-level confirmation bias produces by itself a human tendency to polarized attitudes in societies.

Recently, confirmation bias has been successfully implemented as a mechanism of polarization in different models [33,34]. In each case, authors showed that adding a confirmation bias mechanism to the classical models of opinion dynamics (here the DeGroot model [12] and BCM for bounded confidence model [15]) can induce polarization in simulations. Our model stands out by not requiring any interactions between agents for polarization to emerge, being very simple and tractable, as well as building up from primary mechanisms rooted in the reward system. Reinforcement learning has also been recently used to model polarization from social feedback [19]. This latter model shares the simplicity and tractability of our case described here but relies on a suite of different reward functions that depend on social feedback. Agents are randomly matched with each other and obtain positive (or negative) reward if they share (or not) the same opinion. For polarization to emerge, agents must then engage in social interactions in the environment. Our model does not require any of these features.

In our paradigm, choices are of prime importance. They are the driving force of the biased information processing. In reinforcement learning tasks, subjects accumulate evidence about reward contingencies in a way that favours options that have been selected, but only if this selection is their very own decision. The effect vanishes in case of forced or observed choices [26]. The controllability of one's actions has also been recently linked to an increase of required evidence in order to change one's mind [46]. In our setting, choice is the statement of attitudes at each time step by the agents. These statements are thus the driving force for polarization. From this perspective, our study can contribute a novel view to the debate of whether internet and social medias increase polarization. Online platforms offer an enormous number of opportunities for the person to reinforce their position by simply stating it and collecting random, incidental feedback. Furthermore, the quasi-limitless opportunity and means of expression are consequential opportunities to reinforcing one's attitude in a confirmatory way. Thus, as a platform for repeatedly making arbitrary inconsequential choices (i.e. expressions), the internet provides a most fertile ground for polarization to grow, even if the amplifying effect of social media (e.g. echo chambers) and targeted content manipulation (e.g. filter bubbles) were effectively eradicated by top-down interventions by governments or social media tech giants such as Twitter or Meta. Our study then suggests that social media contribute to polarization both directly and through the additional effect of echo chambers and filter bubbles. Regarding the latter, our findings also add to the personalization-polarization debate [47] which has received mixed evidence so far [48]. Our implementation of filter bubbles is minimal as the rest of the model, and shares the same core principles as other methods that are inferring agents' interests from past behaviours and recommending content in line with it [47]. In our model, filter bubbles simply increase agents’ likelihood of receiving information consistent with their passed opinion expressions. In more complex multi-object environment, recommender system can exhibit other behaviours such as suggesting objects to agents that bear similarity to ones they have previously expressed liking for [33]. In line with models assuming reinforcement of opinions [47,49], we found that filter bubbles increase polarization. Not only agents process new information in a confirmatory manner but also receive, in their filter bubbles, an increasing proportion of confirmatory information compared to disconfirmatory one. This additional effect strengthens *in fine* the opinions' resilience to informational change in the environment.

The repetitive expression of one's attitude is, in our model, mechanistically linked to the increase of the attitude's extreme nature. This mechanism may strike as counter-intuitive. Not to mention the philosophical defense of freedom of expression [50], it is widely thought that increasing citizens' opportunities for unbridled self-expression (think about Hyde Park Corner's iconic place in enlightenment and Western democracy) is beneficial to the well-functioning of democratic societies. Our mechanistic hypothesis challenges this claim and push us to rethink the link between people's expression opportunities and the extreme nature of their attitudes. In our model, each expression opportunity activates and orients one's confirmation bias in a way that amplifies the polarization of their attitudes, whereas listening without expression may not. In fact, results from a previous study [26] indicate that matching another agent's decision instead of making our own does not activate confirmation bias. Further behavioural studies would be needed to test whether expression opportunities are necessary for the emergence and/or amplification of attitude polarization.

Whereas we focused, as many studies before, on the simplest case of two options or poles, our virtual environment can accommodate any number of these, and our results would hold for each of them. In an *n* options environment, agents would eventually prefer one of them, and the attitude map would eventually contain *n* groups. The environment simulated in our study is also extremely simple and flexible computationally, and yet could then easily be adapted to test additional hypotheses about the information delivery, interaction mechanisms or other environmental events.

In recent years, the study of confirmation bias has spread to the analysis of lower-level cognitive processes such as visual perception [22,23] and reinforcement learning [24–26]. Participants in these studies have been found to accumulate visual evidence in random motion dot task and monetary rewards in bandit task, in a choice-dependent fashion. In both types of task, stimuli are neutral in essence and no prior beliefs should play a role in the confirmatory processes. In reinforcement learning, it has been recently shown that the bias allows virtual agents to gather more rewards compared to unbiased learning [27]. This counter-intuitive result is explained by the noisy nature of the decision process in animals in general and humans in particular. Through an extremization of values learned during the task, resilience to noise increases because the best options appears even better than what they actually are and conversely for the worst options. Confirmation biases seem then to be rooted at the core of information processing. Consistent with this notion, a recent MEG study showed that the neural encoding of new evidence is consistent with a selective gating of choice-dependent information [23]. It has also been linked to dopaminergic genes [40] and found to be adaptive when coupled with efficient metacognition [51]. All together, these results tend to indicate that a certain extent of attitude polarization is to be expected and tolerated positively in any population of human subjects who have to make decisions under uncertainty.

The recent implementation of the reinforcement learning paradigm in the study of polarization [19] provided neurobiological grounds to the modelization of opinion dynamics through social feedback [52]. The model presented in the current study similarly attributes opinion polarization to the human reward system, albeit through individual confirmatory feedback. Reinforcement learning has been found to relate on dopaminergic modulation through numerous electrophysiology, pharmacological and neuroimaging studies [53–59] and a recent review [38] suggests that this neural reward system can support asymmetric reward learning in general, and confirmatory updates in particular. On the one hand, positivity bias (i.e. a learning asymmetry favouring positive prediction error over negative ones) has been positively linked to higher striatal activation [24] and to higher dopamine [60–62]. On the other hand, reinforcement learning studies [63,64] with complete feedback (as in our model) show that factual and counterfactual outcomes are both encoded in the striatum, with opposite signs. Taken together, these results suggest that confirmatory updates as implemented in our model, consisting of a positivity bias for factual outcomes and an inverse positivity bias for counterfactual outcomes, are indeed governed by the dopaminergic reward system. Polarization in our model could then find its roots in the very functioning of this system, on which rely general reinforcement learning mechanisms and asymmetric updates. Future studies could strengthens this hypothesis by testing it in a more direct manner, combining our paradigm to a human neuroimaging study.

Confirmation is a broad label that refers to a family of tendencies, and previous work has documented how some of these tendencies could contribute to polarization through beliefs-formation or updating. For instance, confirmation bias shows up as individuals (more than algorithms) are limiting their own exposure to disconfirmatory, ‘cross-cutting’ content [65]. Our framework suggests here that even under equal exposure to confirmatory and disconfirmatory information, polarization occurs and stabilizes due to asymmetrical treatment of both types of information. What makes an agent favouring one of the two objects is the random initial conditions which can locally be advantageous for one of them. We showed that random initialization does not affect the extent of polarization in the population but at the individual level, it randomly directs agents' attitudes towards one object. This result emphasizes the importance of prior beliefs in the individual orientation of subjects towards any given opinion, party or object. At the macro level, initial preferences do not matter and any kind of starting point leads to the same end product, a polarized crowd. At the micro level, though, whatever the random initial attitude individuals are dealt with by any ‘invisible’ hand, they would end up going to a more extreme version of it.

The present study explored the implication of individual confirmatoryoriented treatment of information on attitude polarization at the group level. However, it remains silent on higher-level cognitive processes which are at play in this phenomenon. We describe a tendency rooted in the neural information processing, which play irremediably a role, even minimal, along with those higher level cognitive aspects. And as an independent mechanism contributing to group polarization, it should be dealt with and taken into account in the study of social and political dynamics.

## 4. Methods

### (a) Simulation parameters

Main simulation parameters are presented in the Results section and in figure 1*a*. This subsection give additional details on the simulation parameters. Artificial agents make decision through a *softmax* policy. The probability of making a statement in favour of object 1 over object 2 at time *t* is computed as

$$P_{1}^{t}=\frac{e^{Q_{1}^{t}/\beta }}{e^{Q_{1}^{t}/\beta }+e^{Q_{2}^{t}/\beta }}.$$

Where β is the softmax temperature and set to 0.1 in all simulations. The probability of making a statement in favour of object 2 is computed equivalently.

Unless indicated otherwise, simulations are performed with a moderate level of confirmation bias with $\alpha_{c}=0.3$ and $\alpha_{d}=0.1$.

### (b) Echo chambers

Echo chambers are implemented through an increase in the probability of receiving confirmatory information and an associated decrease of receiving disconfirmatory information. This probability change affects only agents who are *members* of the echo chamber (i.e. who have expressed their attitude in favour of the same object in the last 8 time steps). The probability of receiving confirmatory information is computed as

$$P_{\mathrm{con}}^{t}=P_{\mathrm{prior}}+\left((1-P_{\mathrm{prior}})*\frac{N_{\mathrm{con}}^{t}}{N_{\mathrm{agent}}}\right),$$

and the probability of receiving a disconfirmatory information as:

$$P_{\mathrm{dis}}^{t}=P_{\mathrm{prior}}-\left((1-P_{\mathrm{prior}})*\frac{N_{\mathrm{con}}^{t}}{N_{\mathrm{agent}}}\right),$$

where $P_{\mathrm{prior}}=0.5$ in all simulations performed in the study, $N_{\mathrm{con}}^{t}$ is the number of agents in the echo chamber at time *t*, and $N_{\mathrm{agent}}$ the total number of agents in the environment. Both probabilities are bounded between 0 and 1, and their sum is equal to 1.

### (c) Filter bubbles

Filter bubbles are implemented through a matching of the probabilities of receiving positive information and the agents' proportion of choice towards both objects from the beginning of the simulation. This probability change affects all agents after 8 time steps. The probability of receiving positive information for object 1 is computed as

$$P_{1}^{t}=\frac{N_{1}^{t}}{N_{\mathrm{choice}}^{t}},$$

and the probability of receiving positive information for object 2 as:

$$P_{2}^{t}=\frac{N_{2}^{t}}{N_{\mathrm{choice}}^{t}},$$

where $N_{1}^{t}$ and $N_{2}^{t}$ are the number of statements in favour of objects 1 and 2 respectively, and $N_{\mathrm{choice}}^{t}$ is the total number of statements so far in the simulation. Both probabilities are bounded between 0 and 1, and their sum is equal to 1.

## Data Availability

Data and codes are available upon request to the corresponding author. Supplementary material is available online [66].
